# Musical Preferences are Linked to Cognitive Styles

**DOI:** 10.1371/journal.pone.0131151

**Published:** 2015-07-22

**Authors:** David M. Greenberg, Simon Baron-Cohen, David J. Stillwell, Michal Kosinski, Peter J. Rentfrow

**Affiliations:** 1 Department of Psychology, School of Biological Sciences, University of Cambridge, Cambridge, United Kingdom; 2 Autism Research Centre, Department of Psychiatry, University of Cambridge, Cambridge, United Kingdom; 3 The Psychometrics Centre, Department of Psychology, University of Cambridge, Cambridge, United Kingdom; 4 Department of Computer Science, Stanford University, Stanford, California, United States of America; The University of Chicago, UNITED STATES

## Abstract

Why do we like the music we do? Research has shown that musical preferences and personality are linked, yet little is known about other influences on preferences such as cognitive styles. To address this gap, we investigated how individual differences in musical preferences are explained by the empathizing-systemizing (E-S) theory. Study 1 examined the links between empathy and musical preferences across four samples. By reporting their preferential reactions to musical stimuli, samples 1 and 2 (*Ns* = 2,178 and 891) indicated their preferences for music from 26 different genres, and samples 3 and 4 (*Ns* = 747 and 320) indicated their preferences for music from only a single genre (rock or jazz). Results across samples showed that empathy levels are linked to preferences even within genres and account for significant proportions of variance in preferences over and above personality traits for various music-preference dimensions. Study 2 (*N* = 353) replicated and extended these findings by investigating how musical preferences are differentiated by E-S cognitive styles (i.e., ‘brain types’). Those who are type E (bias towards empathizing) preferred music on the Mellow dimension (R&B/soul, adult contemporary, soft rock genres) compared to type S (bias towards systemizing) who preferred music on the Intense dimension (punk, heavy metal, and hard rock). Analyses of fine-grained psychological and sonic attributes in the music revealed that type E individuals preferred music that featured low arousal (gentle, warm, and sensual attributes), negative valence (depressing and sad), and emotional depth (poetic, relaxing, and thoughtful), while type S preferred music that featured high arousal (strong, tense, and thrilling), and aspects of positive valence (animated) and cerebral depth (complexity). The application of these findings for clinicians, interventions, and those on the autism spectrum (largely type S or extreme type S) are discussed.

## Introduction

Music is a prominent feature of everyday life and a cultural universal [1, 2]. Each day we come across music of varying styles and characteristics, and we continually make judgments about whether or not we like the music we hear. When listening to a new song, it takes us just a few seconds to decide whether to press repeat, change to the next tune, or to buy it. However, little is known about what determines our taste in music. We address this gap in the literature by examining the cognitive and affective underpinnings of musical preferences.

Research over the past decade has argued that musical preferences reflect explicit characteristics such as age, personality, and values [3–6]. Indeed, findings across studies and geographic regions have converged to show that the Big Five personality traits are consistently linked to preferences [6–12]. For example, people who are open to new experiences tend to prefer music from the blues, jazz, classical, and folk genres, and people who are extraverted and agreeable tend to prefer music from the pop, soundtrack, religious, soul, funk, electronic, and dance genres [13].

Though these findings are consistent across studies, what is also consistent is that the results have small effect sizes (*r* < .30) when compared to benchmarks used in other psychological research [14]. This raises the question of whether there are additional psychological mechanisms that might account for individual differences in musical preferences. In this article we build on previous research by examining two dimensions that may be linked musical preferences: empathy and systemizing.

### Empathizing-Systemizing (E-S) Theory and Music Research

Music listening involves a range of abilities. These include: *perceptual processing*: taking in and making sense of audio and visual content in music [15, 16]; *affective reactivity*: reacting emotionally and physiologically to it [17–19]; *intellectual interpretation*: interpreting how the detailed emotional and sonic elements in the music relate to the whole [20]; and *prediction*: anticipating the expected direction of the music (e.g. the melody or narrative) and predicting the thoughts and feelings of the musician [21–24].

These musical abilities may overlap with the drives to empathize and systemize. Empathy is the ability to identify, predict, and respond appropriately to the mental states of others [25, 26]. People use empathy when perceiving musical content, reacting emotionally and physiologically to it, and while performing [27–32]. Systemizing is the ability to identify, predict, and respond to the behavior of systems by analyzing the rules that govern them [33]. These include systems that are natural (e.g. the weather), abstract (e.g. mathematics), organizational (e.g. classifications), and technical (e.g. a mechanical motor) [34, 35]. People are likely to systemize when perceiving and interpreting musical content, particularly when analyzing and deconstructing its sonic features and interpreting how the detailed elements in a musical piece relate to the whole [36]. Even though research into music and empathy has increased, there remains very little empirical research into systemizing and music [37]. This is surprising given that there is evidence that empathy and systemizing are not entirely independent of each other [33, 38–40].

Individual differences in empathy can be measured by the Empathy Quotient (EQ) [26] and systemizing can be measured by the Systemizing Quotient-Revised (SQ-R) [41], and both have contributed to the empathizing-systemizing (E-S) theory [38–39]. Measurements on these two dimensions reveal a person’s cognitive style (or ‘brain type’). Those who score higher on the EQ than the SQ are classified as ‘type E’ (empathizing), and those who score higher on the SQ than the EQ are classified as ‘type S’ (systemizing). Individuals with relatively equal scores on both are classified as ‘type B’ (balanced). Research has provided evidence that these two dimensions explain psychological sex differences. More females are classified as type E and more males are classified as type S [40]. Furthermore, scores on the EQ and SQ predict autistic traits as measured by the Autism Spectrum Quotient (AQ) [41, 42]. Those on the autism spectrum are typically classified as type S or ‘extreme type S’ [41, 43, 44]. These brain types have a neurobiological basis [45, 46]. In males for example, systemizing is positively linked to the size of the hypothalamic and ventral basal ganglia brain regions [47].

There have only been a few studies that have explored how empathy links to musical preferences, and there have been no studies on systemizing. Vuoskoski and colleagues [32] asked participants (*N* = 148) to indicate their liking for 16 musical excerpts from film music. The 16 excerpts were categorized into four groups: sad, happy, scary, and tender. Results showed that empathy was positively correlated to preferences for sad and tender music, and there were no significant correlations for happy or scary music. However, because the excerpts were exclusive to film music, the extent to which these findings generalize beyond the soundtrack genre is not yet known. In another study, Egermann & McAdams [48] found that preferences moderated the relationship between empathy and emotion contagion in music, however, they did not examine the direct links between empathy and individual differences in musical preferences. Therefore, we extend this previous research by using a music-preference model that examines preferences with stimuli representative of the musical variety that people listen to in everyday life, and which also overcomes critical limitations in previous research in the area of musical preferences.

### Methodological Issues

Research into musical preferences has long been hindered by constraints posed by genre-based methodologies. Researchers frequently measure preferences by asking participants to indicate their self-ratings of preferences for a list of genres [6]. However, genres are artificial labels that have been developed over a period of decades by the record industry, and which contain illusive definitions and social connotations. They can hold different definitions depending on the time period that is in reference. For example, the ‘jazz’ label can refer to the swing era of the 1930’s and 40’s and the music of Louis Armstrong and Count Basie, but it can also refer to the post-bop and avant-garde era of the 1960’s and 70’s, which featured the music of John Coltrane and Sun Ra. Genres are also umbrella terms that cover a variety of sub-styles. For example, the ‘rock’ label can refer to ‘soft rock’, such as music by Billy Joel and Elton John, but also ‘hard rock’, such as music by AC/DC and Guns N’ Roses. Therefore, genre-based methodologies that ask participants to indicate their liking for genre labels make it difficult for researchers to accurately capture information about an individual’s preferences.

To address this issue, Rentfrow, Goldberg, & Levitin [49] measured musical preferences across four independent samples by asking participants to report their preferential reactions to musical stimuli that were representative of a variety of genres and subgenres. Separately, judges rated these excerpts based on their perceptions of various sonic (e.g. instrumentation, timbre, and tempo) and psychological (e.g. joyful, sad, deep, and sophisticated) attributes in the music. Findings across all of the samples converged to suggest that a robust five-factor structure underlies musical preferences, and that each of the five dimensions are defined and differentiated by configurations of their perceived musical attributes. These dimensions (coined the MUSIC model after the first letter of each dimension label) are: Mellow (featuring romantic, relaxing, unaggressive, sad, slow, and quiet attributes; such as in the soft rock, R&B, and adult contemporary genres); Unpretentious (featuring uncomplicated, relaxing, unaggressive, soft, and acoustic attributes; such as in the country, folk, and singer/songwriter genres); Sophisticated (featuring inspiring, intelligent, complex, and dynamic attributes; such as in the classical, operatic, avant-garde, world beat, and traditional jazz genres); Intense (featuring distorted, loud, aggressive, and not relaxing, romantic, nor inspiring attributes; such as in the classic rock, punk, heavy metal, and power pop genres); and Contemporary (featuring percussive, electric, and not sad; such as in the rap, electronica, Latin, acid jazz, and Euro pop genres).

We employ the MUSIC model in the current investigation because of four notable advantages. First, the five factors are recoverable not only across genres but also within. In two independent studies, the MUSIC model was replicated within preferences using music from only a single genre [50]. It was first replicated among preferences for jazz music, and second within preferences for rock music. Second, the model has ecological validity because the excerpts administered were of studio recorded music, as opposed computer-generated or manipulated music for the purposes of an experiment. Third, by consulting experts in the field, the musical excerpts were selected via a systematic procedure that aimed to generate a stimulus set that was representative of the large spectrum of musical characteristics and styles that people are exposed to in their everyday lives. Fourth, because each of the excerpts was coded for their sonic and psychological attributes, fine-grained observations about an individual’s musical preferences are able to be made.

### Aims

The aim of this research was to investigate the cognitive and affective basis of musical preferences by asking people to report their preferential reactions to musical stimuli. To address this aim, we used multiple samples, musical stimuli, and recruitment routes to examine how individual differences in musical preferences are empirically explained by empathizing, systemizing, and cognitive ‘brain types’. The specific aims of this research were:

1. To examine if empathizing and systemizing correlates with musical preferences across multiple samples.
2. To test if replicated patterns of results emerge for preferences within a single genre of music: first within rock music and second within jazz music.
3. To examine how individual differences in musical preferences are differentiated by brain type. Specifically, we examined how preferences for broad musical styles (as outlined by the MUSIC model) are differentiated by brain type E, type B, and type S.
4. To examine how preferences for fine-grained features in music (preferences for specific psychological and sonic attributes) vary according to brain type.
5. To test the extent to which the findings are independent of sex and personality traits.

Contemporary research into musical preferences has adopted an interactionist approach posting that people seek musical environments that reflect and reinforce their personal characteristics (e.g. personality traits) [4, 6] Extending this theory to cognitive styles, we predicted that people would prefer music that reflects their empathizing and systemizing tendencies. Because empathizers have a tendency to perceive and react to the emotional and mental states of others, we predicted that empathizers would prefer music that reflects emotional depth. Elements of emotional depth are often heard in the Mellow and Unpretentious music-preference dimensions, which features soft, gentle, reflective, thoughtful and warm attributes [50]. And because systemizers have a tendency to construct and analyze systems, we predicted that systemizers would prefer music that contains intricate patterns and structures. These elements are often heard in the Sophisticated music-preference dimension, which features instrumental, complex, and intelligent or cerebral attributes (ibid). Importantly, since systemizers often have lower levels of empathy, we predicted that systemizers are likely to prefer music opposite to that which is featured in the Mellow dimension, including music with strong, tense, and thrilling, and attributes, which is featured in music from the Intense music-preference dimension.

## Study 1: The Role of Empathy in Musical Preferences

The aim of this study was to examine how empathy levels link to musical preferences across multiple samples and musical stimulus sets. Samples 1 and 2 were administered stimuli from a variety of genres. This allowed us to observe how empathy levels are linked to preferences across different genres. To test the robustness of these patterns, samples 3 and 4 were administered stimuli from only a single genre of music: rock and jazz genres, respectively. This allowed us to observe whether similar patterns of results were recoverable for preferences within only a single genre. Further, because research indicates clear sex differences in empathy levels, we tested if the associations between empathy and musical preferences are independent of sex.

Previous research has shown across both behavioral and self-report measures that musical preferences are linked to the Big Five personality traits [6]. And, some researchers [51, 52] have argued that there are overlaps between empathizing and dimensions of the Big Five (particularly Agreeableness). Therefore, we tested the extent to which associations between empathy and musical preferences are independent of the Big Five.

## Method

The data presented in this study are based on four distinct samples. Participants in each sample were recruited via Facebook through the myPersonality Facebook application [53]. Through this application, Facebook users were able to complete a variety of psychology-related questionnaires. After completing a questionnaire, participants received instant feedback about their scores and were given the option of posting them on their personal Facebook profiles for other users to view. Each sample completed the same empathy measure but they differed in the musical stimuli presented to them. Participants completed the empathy and music-preference measures on separate occasions. Details about each sample are described below and S1 Table provides a summary of the sample characteristics for each of the four samples. The data from these samples are secondary, anonymized, previously published in the public domain (http://mypersonality.org/), and were originally gathered with an explicit opt-in consent for reuse for research purposes that extend beyond the original project. Accordingly, the Psychology Research Ethics Committee of the University of Cambridge confirmed that no IRB approval was needed.

### Participants

Only participants who responded to all of the items were included in the final analysis.

- Sample 1 (S1) included 2,178 participants. Of the 2,015 (93%) who indicated their sex, 1,200 (60%) were female and 815 (40%) were male. The sample ranged from 18 to 59 with the mean age = 24.80 (*SD* = 7.50).
- Sample 2 (S2) included 891 participants. Of the 807 (91%) who indicated their sex, 512 (63%) were female and 295 (37%) were male. The sample ranged from 18 to 58 with the mean age = 23.71 (*SD* = 6.47).
- Sample 3 (S3) included 747 participants. Of the 708 (95%) who indicated their sex, 423 (60%) were female and 285 (40%) were male. The sample ranged from 18 to 54 with the mean age = 25.31 (*SD* = 7.21).
- Sample 4 (S4) included 320 participants. Of the 297 (93%) who indicated their sex, 169 (57%) were female and 128 (43%) were male. The sample ranged from 18 to 61 with the mean age = 24.63 (*SD* = 7.45).

### Measures

#### Empathy

Participants in each sample completed the Empathy Quotient (EQ) [26]. The EQ is a 60-item self-report questionnaire that measures cognitive and affective components of empathy. 20 of the 60 items are filler leaving a total of 40 items that measure empathy directly. Participants are required to indicate their degree of agreement for each statement on a four point scale (*strongly disagree*, *slightly disagree*, *slightly agree*, or *strongly agree*). For positively poled items, two points are given for strong agreement and one point is given for slight agreement. For negatively poled items, two points are given for strong disagreement and one point is given for slight disagreement.

#### Personality Traits

Personality in each of the four samples was assessed with a proxy version of the NEO Personality Inventory-Revised [54] developed by the International Personality Item Pool [55]. The NEO-PI-R is a widely used and accepted instrument to assess personality as conceptualized by the Five Factor Model (FFM) [56]. The FFM groups traits hierarchically into five broad factors: Neuroticism (N), Extraversion (E), Openness to Experience (O), Agreeableness (A), and Conscientiousness (C) [57]. Each of these five factors is broken down into six facets. Reliabilities between the IPIP proxy and the NEO Personality Inventory range from .71 (dutifulness) to .92 (anger) (http://ipip.ori.org/). Participants had the option to complete a 336-item version or a shortened 20- to 100-item version of the IPIP proxy. The latter version was administered to participants in 10-item blocks and participants determined the amount of questions to complete after finishing each 10-item block. The majority of participants completed the full 100-item version. For example in S1, 1,371 (63%) participants completed the 100-item version, 510 (23%) completed the 336-item version, 167 (8%) completed the 20-item version, 65 (3%) completed the 30- to 90-item versions, and 65 (3%) did not complete any measure of personality.

#### Musical Stimuli

We administered the same music stimulus sets that have been used as in previous research on musical preferences [49, 50]. Importantly, to reduce confounds related to participants’ previous exposure and associations with the musical stimuli, the excerpts were selected from Getty Images, from which the copyright for the music was purchased. Therefore, it was highly unlikely that the participants had previous exposure to any of the musical excerpts. Participants were informed that the musical preference measure would require them to listen to 15-second long musical excerpts using speakers or headphones. Participants were then presented with the excerpts and asked to report their degree of liking for each. S1 was administered 50 excerpts that represented 26 different genres and subgenres [50]. A subset of 25 of these mixed genre excerpts was administered to S2 (this subset is the same as that used in previous research where they were selected not only based on factor loadings, but also on the extent to which they represented the breadth of the five factors of the MUSIC model) [49]. In S3, participants were administered 50 excerpts from only the rock genre [50]. S4 was administered 50 excerpts from only the jazz genre [50]. The musical excerpts used in each of the samples are listed in S2 Table in the online material.

### Statistical Analysis

We assessed musical preferences by calculating each participant’s weighted preference scores for each of the five MUSIC dimensions. Specifically, to calculate the mean preference score for each dimension, we multiplied the participant’s rating for each excerpt by the excerpt’s factor loading (standardized using Fisher’s *r*-to-*z* transformation) on the dimension in question (e.g. Mellow); factor loadings for each excerpt were used from factor analyses previously reported [50]. To calculate the average weighted preference for each participant across the excerpts, we then added the weighted preference of each excerpt and divided that sum by the sum total of preference ratings for all of the excerpts presented to them. We conducted this calculation for each of the five music-preference dimensions. As an example, below is the formula used for calculating the weighted preference rating for the Mellow dimension.

$$\text{Weighted Preference for the Mellow Dimension = }\frac{\begin{array}{l} {}[(\text{preference for excerpt 1) * (excerpt 1's loading onto the Mellow dimension)] +}\\ \text{[(preference for excerpt 2) * (excerpt 2's loading onto the Mellow dimension)] + }\ldots \\ {}[(\text{preference for excerpt 50) * (excerpt 50's loading onto the Mellow dimension)]} \end{array}}{(\text{Sum of preference ratings for all 50 excerpts)}}$$

### Results and Discussion

#### Descriptive statistics

The maximum score possible on the EQ is an 80. Across the four samples, mean scores on the EQ ranged from *M* = 41.89 (*SD* = 13.68) (S2) to *M* = 44.03 (*SD* = 12.8) (S3). Analysis of variance (ANOVA) revealed a main effect of sex in each of the four samples: F(1, 2013) = 71.45, *p* < .01; F(1, 805) = 27.70, *p* < .01; F(1, 706) = 17.05, *p* < .01; and F(1, 295) = 7.98, *p* < .01), for S1, S2, S3, and S4, respectively. Females scored higher than males in each of the samples. Mean scores on the EQ for females ranged from *M* = 43.71 (*SD* = 12.94) (S2) to *M* = 45.59 (*SD* = 12.22) (S3) and mean scores for males on the EQ ranged from *M* = 38.63 (*SD* = 13.62) (S2) to *M* = 41.56 (*SD* = 13.47) (S3). Reliability calculated using Cronbach’s alpha was high in each of the samples (S1 = .90, S2 = .91, S3 = .89, and S4 = .90).

#### Empathy correlates of musical preferences

Table 1 reports correlation coefficients between the EQ and musical preferences across the four samples. There are several general and specific findings. In general, the magnitudes of the correlation coefficients are small, but they are consistent with those found in other studies on the correlates of musical preferences, which generally report smaller correlations when compared to benchmarks used in other psychological research [13]. Importantly, the strongest correlations between EQ and musical preferences across the four samples are for the Mellow and Intense music-preference dimensions.

**Table 1:** Correlations Between the Empathy Quotient and Musical Preferences.

	Empathy Quotient			
	S1	S2	S3	S4
	(Mixed Genre)	(Mixed Genre)	(Rock Music)	(Jazz Music)
**Mellow**	.09**	.11**	.14**	.06
**Unpretentious**	.08**	.04	.04	.01
**Sophisticated**	.03	.01	.00	-.14*
**Intense**	-.10**	-.11**	-.13**	-.08
**Contemporary**	.04*	.09**	.13**	.11*

*Note*. Cell entries are correlations between the Empathy Quotient (EQ) and the MUSIC music-preference dimensions. S1 = Sample 1, S2 = Sample 2, S3 = Sample 3, S4 = Sample 4. S1 and S2 provided preference ratings for mixed genre excerpts; S3 provided preferences ratings for only rock excerpts; and S4 provided preference ratings for only jazz excerpts. *Ns =* 2,178 (S1), 891 (S2), 747 (S3), 320 (S4).

*p < .05;

**p < .01.

Specifically, columns 1 and 2 report correlations based on preferences across mixed genres. In both S1 and S2, EQ is positively linked to the Mellow, Unpretentious, and Contemporary dimensions, and is negatively linked to the Intense dimension. Column 3 reports correlation coefficients from S3 who provided preferences for only rock music excerpts. As can be seen, EQ is positively linked to Mellow rock and Unpretentious rock dimensions and negatively linked to the Intense rock dimension. The results from S3 are largely consistent with those based on preferences for mixed genres found in S1 and S2. To further examine the extent to which EQ is correlated with preferences within a single genre, column 4 reports correlation coefficients from S4 who provided preferences for only jazz music. As can be seen, EQ is positively linked to the Mellow jazz and Unpretentious jazz dimensions and negatively linked to the Sophisticated jazz and Intense jazz dimensions. With the exception of the Sophisticated jazz dimension, these results are again consistent with the patterns found in S1, S2, and S3. A close examination of the Sophisticated jazz dimension reveals that it shares many of the same musical properties as the Intense dimension from mixed genres, and the Intense rock dimension. For example, as reported in Rentfrow et al. [50], Sophisticated jazz is highly correlated with fast tempo (*r* = .59), strong (*r* = .55), and aggressive (*r* = .56) features. These patterns are unique to Sophisticated jazz and are not present in the Sophisticated dimensions from mixed genres and Sophisticated rock dimensions. This suggests that the high negative correlation between the EQ and Sophisticated jazz is a further indication of a link between EQ and intense music features.

To empirically test the generalizability of these results across the samples we calculated column-vector correlations between the samples for each of the five music-preference dimensions. First, we transformed the correlation coefficients in Table 1 using Fisher’s *r*-to-*z* formula. Second, we correlated the transformed coefficients between each of the samples, which resulted in six column-vector correlations. The column-vector correlations were .91 (S1 and S2), .87 (S1 and S3), .50 (S1 and S4), .99 (S2 and S3), .72 (S2 and S4), and .78 (S3 and S4), with a mean = .79. This is considerably high when taking into account that each of the samples received varied stimulus sets, particularly S3 and S4 who received music from only a single genre. These results indicate that the correlations between empathy and musical preferences have high generalizability across samples and musical genres. S3 and S4 Tables report results from a subsampling analysis in which we performed the same correlational analyses within random subsets of the larger samples (S1 and S2) equating for the smaller samples (S3 and S4).

#### Sex differences

Research has provided clear evidence that there are significant sex differences in empathy levels. Females on average score higher on the EQ than males (Baron-Cohen, 2003), and as previously reported, we found similar trends in the four samples in this study. Therefore, we investigated the extent to which the correlations between musical preferences and empathy are independent of sex. Toward that end, we conducted partial correlations for the EQ and music-preference dimensions while controlling for sex. Partial correlations for the four samples are reported in S5 Table. As can be seen, the correlations between empathy and musical preferences across samples retain their strength and direction for all of the music-preference dimensions when controlling for sex. These findings suggest that the associations between empathy and musical preferences are robust and independent of sex differences.

#### Personality

Some researchers have argued that empathy (as specifically measured by the EQ) is not distinct from the Big Five personality traits [51, 52]. Therefore, we sought to test the extent to which the links between empathy and musical preferences are independent from the relationship between the Big Five and musical preferences. We performed partial correlations for the EQ and the music-preference dimensions while controlling for scores on the five personality domains. Partial correlations for the four samples are reported in S3 Table. As can be seen, when controlling for personality, the correlations between empathy and musical preferences across samples retain their strength and direction for all of the music-preference dimensions. These findings suggest that the associations between empathy and musical preferences are robust and independent of the links between preferences and personality.

We then sought to examine the extent to which EQ predicts musical preferences over and above the Big Five. To address this issue we tested the incrementally validity of the EQ as a predictor of musical preferences. Specifically, in each of the four samples we regressed each of the five music-preference dimensions onto Big Five scores at Step 1 and EQ scores at Step 2. The results from these analyses are reported in Table 2 and indicate the amount of variance in musical preferences which is explained by personality and empathy. As can be seen, EQ increased the variance significantly for 10 of the 20 regressions. In particular, EQ increased the variance significantly for the Mellow and Intense music-preference dimensions across all of the samples except for S4. In S4, however, EQ did increase the variance significantly for the Sophisticated dimension. Also noteworthy is that EQ increased the variance significantly for the Unpretentious dimension in S1 and the Contemporary dimensions in S2 and S3. These results indicate that both personality and empathy play a significant role in predicting musical preferences, however, it raises the question of whether empathy accounts for more unique variance than personality, namely for the Mellow and Intense dimensions.

**Table 2:** Incremental Changes in Multiple Correlations of Music-Preference Dimensions with Personality and EQ as Simultaneous Predictors.

	Music-Preference Dimension																			
	Mellow				Unpretentious				Sophisticated				Intense				Contemporary			
	S1	S2	S3	S4	S1	S2	S3	S4	S1	S2	S3	S4	S1	S2	S3	S4	S1	S2	S3	S4
**Step 1: Personality**	.14	.21	.12	.19	.07	.17	.08	.17	.14	.12	.12	.26	.11	.13	.14	.17	.09	.15	.02	.25
**Step 2: EQ**	.15	.22	.16	.20	.10	.17	.08	.17	.14	.12	.12	.29	.13	.16	.17	.18	.09	.17	.04	.26
**Δ*F***	9.53**	6.38*	8.25**	.66	9.56**	.62	.22	.32	.00	.03	.22	4.94*	8.64**	6.17*	7.15**	1.56	1.41	6.20*	12.23**	1.95
**Step 1: EQ**	.08	.10	.15	.04	.08	.05	.04	.03	.04	.00	.01	.14	.11	.11	.13	.10	.04	.10	.13	.13
**Step 2: Personality**	.15	.22	.16	.20	.10	.17	.08	.17	.14	.12	.12	.19	.13	.16	.17	.18	.09	.17	.19	.26
**Δ*F***	7.12**	7.00**	.57	2.67*	1.24	4.66**	.66	1.77	7.62**	2.34*	2.02	4.12**	2.24*	2.21	1.50	1.49	2.85*	3.46**	3.23**	3.53**

*Note*: Cell entries are multiple *R*s derived from stepwise regressions in which the music-preference dimensions were regressed onto the Big Five personality dimensions and the Empathy Quotient (EQ) in each of the four samples in Study 1. *Ns* = 2,113 (S1), 855 (S2), 739 (S3), and 313 (S4).

*p < .05;

**p < .01.

To address this question, we conducted an additional set of hierarchical regressions across the four samples. Here, the five music-preference dimensions were regressed onto EQ scores at Step 1 and Big Five scores at Step 2. To determine the extent to which EQ accounted for more unique variance than personality, we compared the Δ*F*s from the first set of regressions displayed in the third row where EQ was added at Step 2 (Δ*F*s ranging from .00 to 12.23), to the Δ*F*s from the second set of regressions displayed in the sixth row where personality was added at Step 2 (Δ*F*s ranging from .57 to 7.62). As can be seen, the Δ*F* was greater for EQ across 10 of the 20 comparisons across samples. The most consistent finding across the samples is that EQ accounted for more unique variance in preferences for the Mellow and Intense dimensions. There is added robustness to this finding when considering that there were more personality variables (5) than empathy variables (1) in the regression analyses, and that more predictors typically increase the resulting multiple correlation. Zero-order correlations presented individually for each of the Big Five personality traits and music-preference dimensions are reported elsewhere (Greenberg et al., under review).

#### Summary

The results from Study 1 suggest that the links between empathy and musical preferences are consistent across all four samples. These patterns are not only present across a mixture of genres, but they are also recoverable within preferences for only a single genre of music. Examination of the largest correlation coefficients across the four samples suggests that correlations between empathy and the Mellow and Intense music-preference dimensions are most robust. Addressing concerns that these correlations were driven by sex differences, partial correlations revealed that these links are independent of sex. Importantly, to address concerns argued by previous research that empathy is indistinguishable from personality traits, partial correlations revealed that the links between empathy and preferences are independent of the Big Five, and hierarchical regression analyses revealed the empathy consistently predicted musical preferences and accounted for more unique variance than did personality for the Mellow and Intense music-preference dimensions. Taken together, these results suggest that empathy plays an important role in musical preferences and that other dimensions of the mind related to empathy such as systemizing warrant investigation in relation to preferences.

## Study 2: Musical Preferences and Cognitive Styles

The aim of this study was to extend the findings from Study 1 to the systemizing and cognitive ‘brain type’ components of the empathizing-systemizing theory. In the present study, by assessing scores on both empathy and systemizing measures, observations about brain type were made. Specifically, we examined how musical preferences vary according to brain type E, type B, and type S. First, we examined preferential differences for broad music styles as defined by the MUSIC dimensions. Second, we examined preferential differences for more fine-grained characteristics in music by examining preferences for specific psychological and sonic attributes featured in the music.

## Method

### Participants and Procedures

Participants were recruited via Amazon’s Mechanical Turk (MTurk). Those who agreed to participate were directed to an online survey hosted by Qualtrics. 353 participants completed all measures of the survey. 220 (62%) were female and 133 (38%) were male, and the sample ranged in age from 18 to 68 with the mean age = 34.10 (*SD* = 12.27). This research was given ethical approval by the Psychology Research Ethics Committee of the University of Cambridge in August 2013.

### Measures

#### Empathy and systemizing

Participants completed the same 60-item EQ measure as administered in Study 1 of this paper. Systemizing was measured with the 75-item Systemizing Quotient-Revised (SQ-R) [26]. Participants were required to indicate their degree of agreement for each statement on a four point scale (*strongly disagree*, *slightly disagree*, *slightly agree*, or *strongly agree*). For positively poled items, two points are given for strong agreement and one point is given slight agreement. For negatively poled items, two points are given for strong disagreement and one point is given for slight disagreement.

#### Cognitive ‘brain type’ calculation

We used the same procedures reported in Wheelwright et al. [41] to calculate each of the five brain types outlined in the E-S theory [40]. Brain type is determined by each individual’s *D* score, which is a measure of the standardized difference of their EQ and SQ-R scores. *D* scores are calculated by first standardizing each individual’s EQ and SQ-R raw score by subtracting the typical population mean from each individual score, and then dividing that sum by the maximum possible score (we used the same means that were used in previous research) [41]. The formula that represents this calculation is as follows:

S = (SQ-R - <SQ-R>)/150 and E = (EQ - <EQ>)/80

We then rotated the original EQ and SQ-R axis by 45° to produce two new variables, *D* and *C*. That is, to calculate variable *D*, we subtracted each participant’s E score from their S score and divided this sum by two. To calculate variable *C*, we subtracted their S score from their E score and divided this sum by two. This procedure is defined by the following formula:

D = (S - E)/2 and C = (E - S)/2

*D* scores describe an individual’s tendency to systemize by indicating the difference between their EQ and SQ-R scores. Conversely, *C* scores describe an individual’s tendency to empathize.

Based on their *D* score, those who score between the 2.5^th^ and 35^th^ percentile (E > S) are classified as type E, those who score between the 65^th^ and 97.5^th^ (S > E) percentile are classified as type S, and those who score between the 35^th^ and 65^th^ percentile are classified as type B (i.e., relatively equal E and S scores). Those who score in the lowest 2.5% (E >> S) are classified as extreme type E, and those who score in the highest 2.5% (S >> E) are classified as extreme type S. In the present study, 59 participants were identified as type E, 103 as type B, and 182 as type S. 9 participants were classified as extreme type S and there were no participants that were classified as extreme type E. Therefore, when conducting analyses on musical preferences by differences in brain type, we excluded participants classified as extreme types from the analysis. It is important to note that the distribution in the present sample is skewed toward systemizing when compared to brain type distributions found in previous research [41]. S6 Table shows the percentage of participants with each brain type in the present study compared to those found in previous research. A reason for this skew toward systemizing may be the nature of the MTurk participant pool. It could be argued that an MTurk worker (i.e. an individual who chooses to complete surveys and work-based tasks as a form of income) is likely to have more systemizing tendencies than the general population. Though there is no previous research that has tested this hypothesis, it is important to be cautious when generalizing the results of the present study to other populations.

#### Musical stimuli

Based on concerns about participant fatigue due to the additional SQ-R questionnaire, we administered the same 25 excerpts used in S2 of Study 1 in this paper, rather than the full 50 in S1.

#### Musical attributes

We intended to make detailed observations about musical preferences that go beyond observations about preferences for broad styles. Therefore, we examined preferences for fine-grained musical attributes featured in the excerpts. These musical attributes include two types: 1) *psychological attributes* such as tension, depth, warmth, complexity, and joyfulness, and 2) *sonic attributes* such as instrumentation and timbre that underpin the psychological attributes. Judges ratings of each of the excerpts based on these attributes were previously collected and reported [49, 50]. Here we include 25 psychological attributes that outline arousal, positive and negative valence, and depth in music, and 20 sonic attributes that outline instrumentation and acoustic elements in music. These attribute groupings were determined based on multiple factor analytic studies that have been previously conducted in our lab.

### Statistical Analysis

To calculate preferences for the five MUSIC dimensions, we used the same formula that was used in Study 1 of this paper. We then conducted the same procedure to calculate each individual’s weighted preference for each of the psychological and sonic attributes, however, rather than using factor scores in the formula, we used each excerpt’s mean rating of the psychological or sonic attributes. Specifically, to calculate the mean preference score for each attribute, we multiplied the participant’s rating for each excerpt by the excerpt’s mean rating of the specific attribute in question (e.g. percussive). To calculate the average weighted preference for each participant across the excerpts, we then added each weighted preference of each excerpt and divided that sum by the total sum of preference ratings across all of the excerpts presented to them. We conducted this calculation for each of the psychological and sonic attributes. As an example, below is the formula used to calculate the weighted preference for the percussive attribute.

$$\text{Weighted Preference for the Percussive Attribute = }\frac{\begin{array}{l} {}[(\text{preference for excerpt 1) * (excerpt 1's mean percussive rating)] +}\\ \text{[(preference for excerpt 2) * (excerpt 2's mean percussive rating)] + }\ldots \\ {}[(\text{preference for excerpt 25) * (excerpt 25's mean percussive rating)]} \end{array}}{(\text{Sum of preference ratings for all 25 excerpts)}}\;$$

### Results and Discussion

#### Descriptive statistics

The maximum score possible on the EQ is an 80 and the maximum score possible on the SQ-R is 150. Analysis of variance (ANOVA) revealed a main effect of sex on EQ (F(1, 351) = 32.82, *p* < .01), with females (*M* = 44.43, *SD* = 11.79) scoring higher than males (*M* = 36.98, *SD* = 11.94). There was no significant main effect of sex on the SQ-R (F(1, 351) = 1.56, *p* = .21), but males (*M* = 65.63, *SD* = 19.75) did have a higher average than females (*M* = 62.85, *SD* = 19.75). Reliability using Cronbach’s alpha was high for both the EQ (.88) and SQ-R (.90).

#### Empathy and systemizing correlates of musical preferences

Correlations between musical preferences and scores on the EQ, SQ-R, *D*, and *C* scores are reported in S3 Table. There are four main findings from the correlational analyses. First, the empathy correlates with the MUSIC model are consistent to those found in Study 1. Second, the systemizing correlates are in the opposite direction of the empathy correlates across the MUSIC dimensions and the psychological and sonic attributes. Third, correlations between *C* and *D* scores and musical preferences are about twice as large in magnitude as the correlations with the EQ and SQ-R scores, indicating that the overarching cognitive styles are stronger links than each of the EQ and SQ-R scores is individually. Fourth, consistent with Study 1, these patterns of associations are independent of sex as indicated by results from partial correlations controlling for sex. Correlations between musical preferences and fine-grained psychological and sonic attributes observed across the four samples in Study 1 are also reported in S3 Table.

#### Cognitive ‘brain type’ and preferences for the MUSIC model

**Fig 1:**
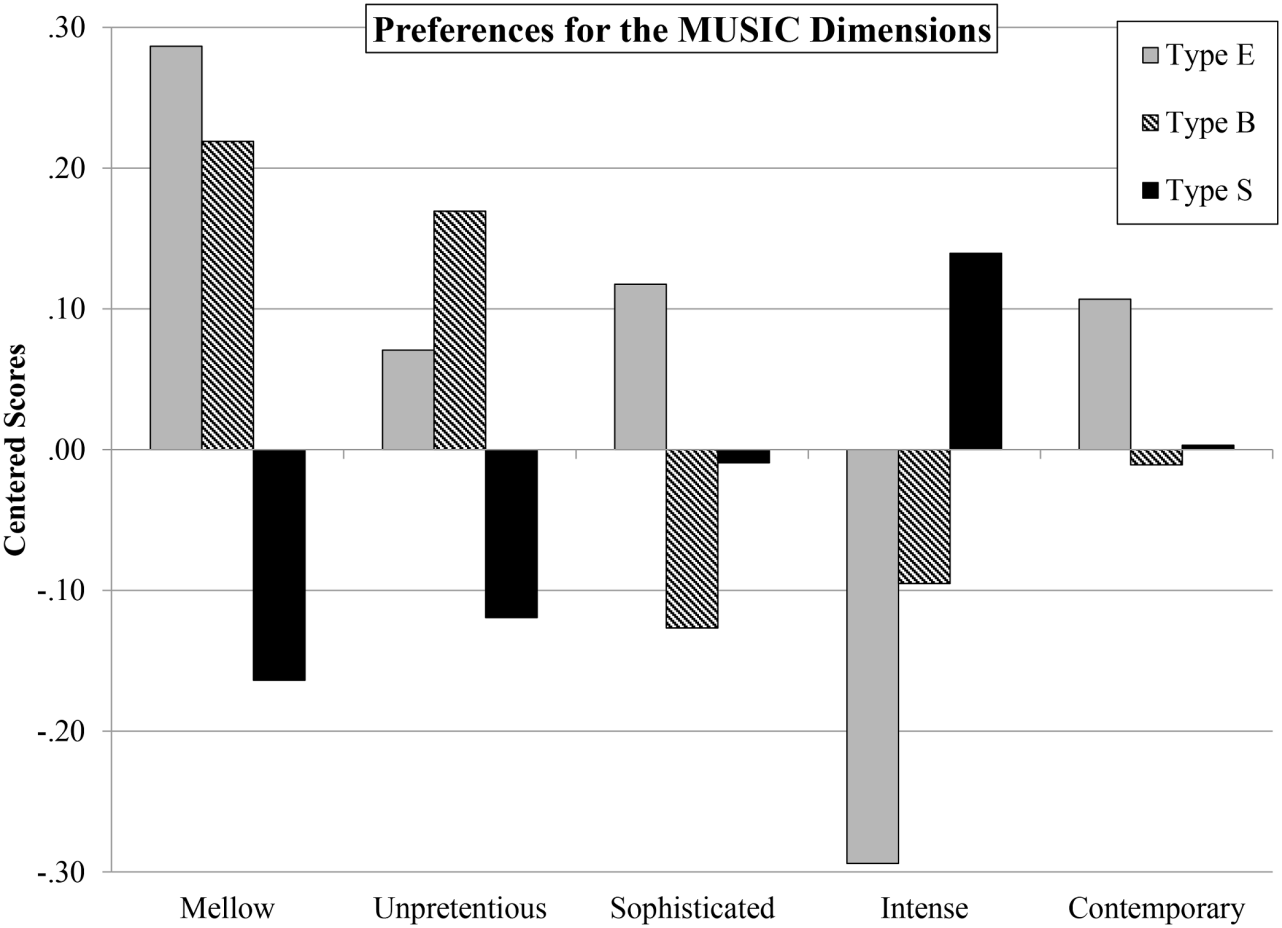
Mean Preferences for the MUSIC Dimensions by Cognitive ‘Brain Type’. For each brain type (Type E, Type B, and Type S), centered mean scores are presented for preferences for each of the five MUSIC dimensions.

To investigate the extent to which musical preferences differ by brain type, we performed analyses of variance (ANOVA) on each of the MUSIC music-preference dimensions using brain type as the independent variable. There was a significant effect of brain type on preferences for the Mellow (F(2, 341) = 7.73 *p* < .01, partial eta squared = .04), and Intense (F(2, 341) = 4.80 *p* < .01, partial eta squared = .03) dimensions. Post-hoc Tukey tests revealed that for the Mellow dimension, type E and type S were significantly different from each other (*p* < .01), and type S and type B were significantly different (*p* < .01). For the Intense dimension, type E and type S were significantly different (*p* < .05). These patterns of results and significance levels remained the same when conducting ANCOVA’s controlling for sex. Fig 1 displays mean differences among brain types for each of the five MUSIC music-preference dimensions. As can be seen, individuals who are type E prefer music from the Mellow dimension and individuals who are type S prefer music from the Intense dimension. S1 Fig displays mean differences in preferences with error bars indicating the standard error. Though the intervals are wide, the error bars for Type E and Type S for Mellow and Intense preferences do not overlap, further suggesting that the differences in preferences between the two are significant.

#### Cognitive ‘brain type’ and preferences for psychological attributes

To investigate the extent to which preferences for specific psychological attributes in music differ by brain type, we performed analyses of variance on each of the 25 psychological attributes (standardized) using brain type as the independent variable. Results revealed a signifcant effect of brain type on preferences for all but three of the psychological attributes (i.e., joyful, fun, and undanceable). Of those for which there was a significant effect, effect sizes ranged from F(2, 341) = 3.68, *p* < .05, partial eta squared = .02 (for amusing) to F(2, 341) = 8.11, *p* < .001, partial eta squared = .05 (for animated). Post-hoc Tukey tests revealed that consistently, type E and type S were significantly different across preferences for the 23 attributes, whereas differences between type E and type B, and conversely, type B and type S, were less frequently significant. These patterns of results and significance levels remained the same when conducting ANCOVA’s controlling for sex, except for five of the attributes which dropped below the level of significance: amusing (F(2, 341) = 1.92, *p* = .15), sophisticated (F(2, 341) = 2.26, *p* = .11), deep (F(2, 341) = 2.64, *p* = .07), not party music (F(2, 341) = 2.42, *p* = .09), and emotional (F(2, 341) = 2.56, *p* = .08).

**Fig 2:**
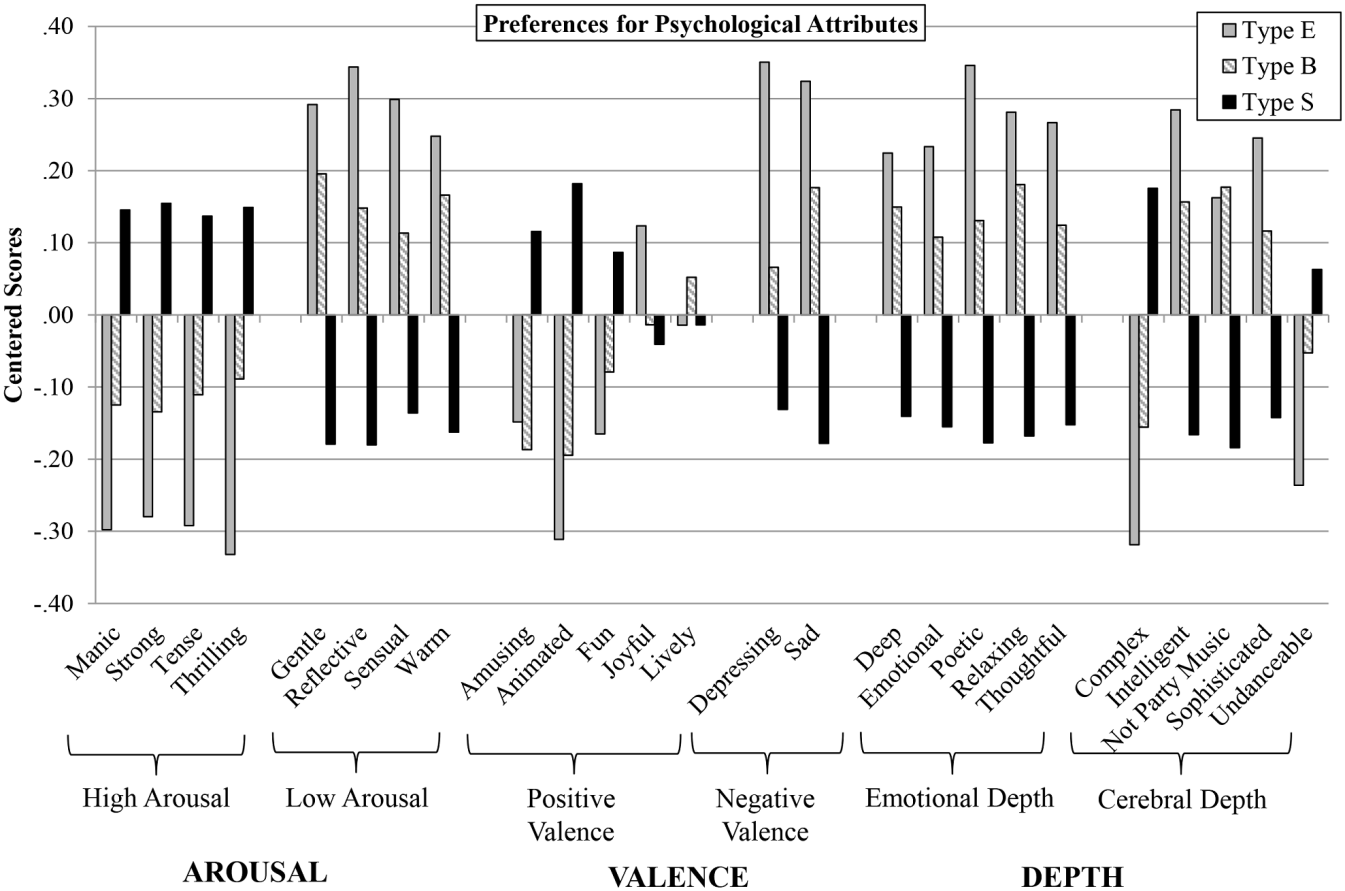
Mean Preferences for Psychological Attributes by Cognitive ‘Brain Type’. For each brain type (Type E, Type B, and Type S), centered mean scores are presented for preferences for 25 psychological attributes. These attributes are categorized into Arousal (high arousal and low arousal), Affect (positive valence and negative valence) and Depth (emotional depth and cerebral depth).

Fig 2 displays mean differences among brain types for preferences for each of the 25 psychological attributes measured in the music. There are clear differences between type E and type S for psychological attributes across the attributes, and in general their preferences map in the opposite direction from each other. Specifically, those who are type S preferred music that featured high arousal (manic, strong, tense, and thrilling attributes), while those who are type E preferred music that featured low arousal (gentle, reflective, sensual, and warm attributes). Type S preferred music that featured positive valence (animated and fun attributes) while type E preferred music that featured negative valence (sad and depressing attributes). In terms of Depth, type E preferred music that featured cerebral depth (intelligent) except for the complex attribute, which type S preferred. Type E also preferred music that featured emotional depth (poetic, relaxing, and thoughtful attributes).

#### Cognitive ‘brain type’ and preferences for sonic attributes in music

To investigate the extent to which preferences for sonic attributes in music differed by brain type, we performed analyses of variance on each of the 20 sonic attributes (standardized) using brain type as the independent variable. Results revealed a significant effect of brain type on preferences for 13 of the 20 sonic attributes (i.e., all except for heavy bass, acoustic guitar, cymbals, piano, raspy voice, woodwinds, and yelling voice). Of those for which there was a significant effect, effect sizes ranged from F(2, 341) = 3.52, *p* < .05, partial eta squared = .02, (strings), to F(2, 341) = 8.76, *p* < .01, partial eta squared = .05 (electric guitar). Post-hoc Tukey tests revleaed that consistently, type E and type S were significantly different across preferences for the 13 attributes, whereas differences between type E and type B, and conversely, type B and type S, were less frequently significant. These patterns of results and significance levels remained the same when conducting ANCOVA’s controlling for sex, except for four of the attributes which dropped below the level of significance: instrumental (F(2, 341) = 2.69, *p* = .07), electric (F(2, 341) = 2.63, *p* = .07), bass guitar (F(2, 341) = 2.59, *p* = .08), and cymbals (F(2, 341) = 1.76, *p* = .17).

**Fig 3:**
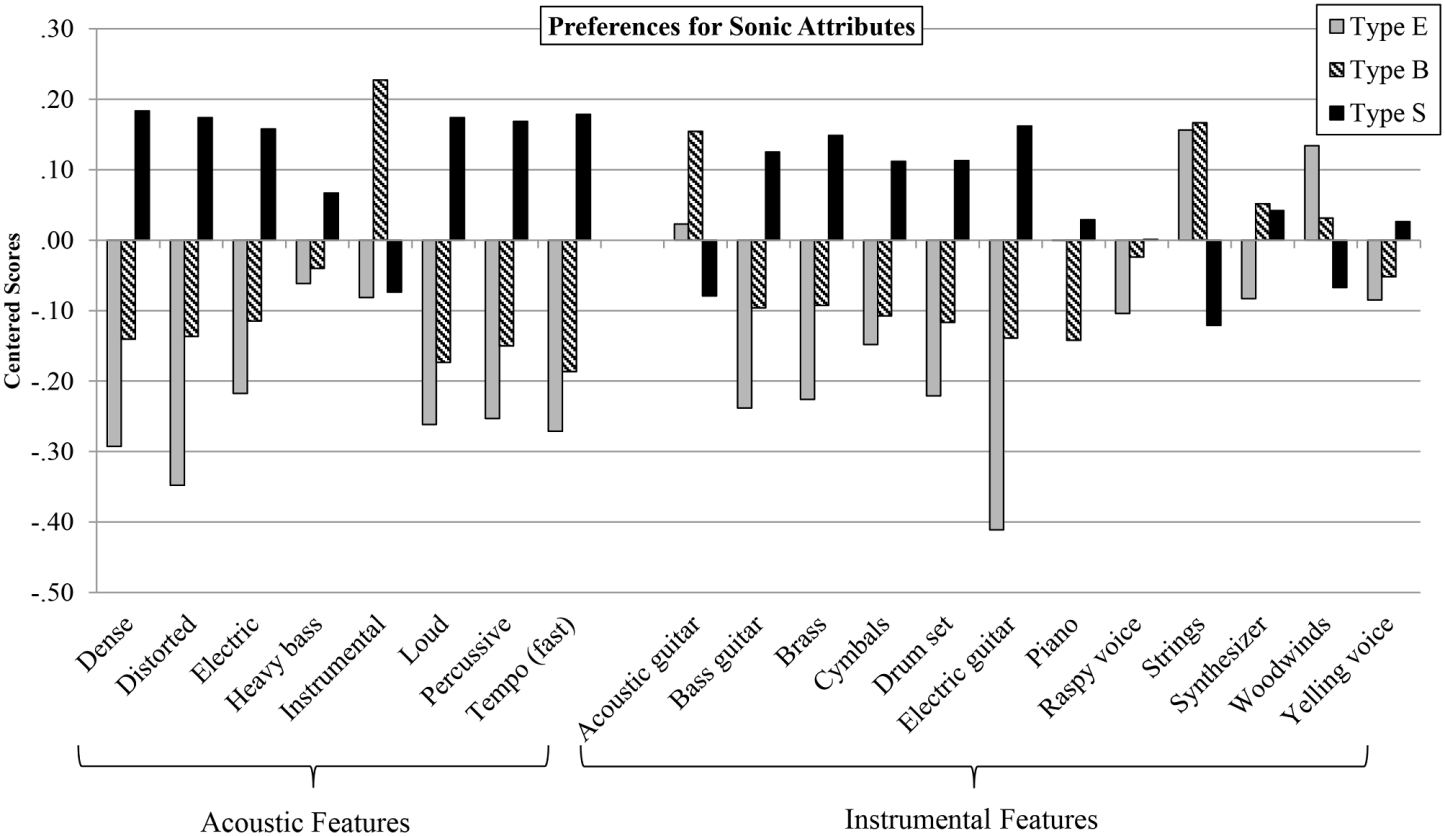
Mean Preferences for Sonic Attributes by Cognitive ‘Brain Type’. For each brain type (Type E, Type B, and Type S), centered mean scores are presented for preferences for 20 sonic attributes. These attributes are categorized into acoustic features and instrumental features.

Fig 3 displays mean differences among brain types for preferences for each of the 20 sonic attributes measured in the music. As can be seen, those who are type S preferred music with acoustic features that were dense, distorted, loud, percussive, and fast in tempo. In terms of instrumental features, type S preferred music that featured brass and electric guitar, while those who are type E preferred music that featured strings.

#### Summary

The results from Study 2 replicate the findings on empathy and musical preferences from Study 1 and extend them to show that systemizing is also linked to musical preferences. Specifically, empathizing and systemizing scores correlated with musical preferences in opposite directions. When calculating the difference between participants’ empathy and systemizing scores, correlations between the resulting brain type scores (*C*/*D* scores) and musical preferences were nearly double in magnitude than the empathy and systemizing scores were individually.

Results revealed that musical preferences for broad musical styles (the MUSIC dimensions) differed by E-S cognitive ‘brain types’. Because the excerpts were rated on perceptions of their musical attributes, we were able to make detailed observations about the nuanced musical characteristics that people prefer. Specifically, preferences for both psychological and sonic attributes featured in the music were found to differ by brain type. As in Study 1, the findings from Study 2 were independent of sex differences. Taken together, these results strongly suggest that cognitive styles underpin individual differences in musical preferences. The findings also support previous evidence that suggest empathy and systemizing are related constructs [33, 38–40]. Therefore, considering that previous research into music and empathy has largely neglected systemizing, future research on the topic would benefit from studying both empathy and systemizing in tandem.

## General Discussion

### Summary of Findings

We investigated cognitive and affective components that are linked to musical preferences. Rather than relying on genre-based methodologies, information about musical preferences were gained by having participants report their preferential reactions to a variety of musical stimuli. Findings from Study 1 revealed that across genres, empathy levels were positively correlated with preferences for Mellow music (R&B/soul, adult contemporary, soft rock genres) and negatively correlated with preferences for Intense music (punk, heavy metal, and hard rock genres). Study 1 also tested if these results could be replicated within preferences for a single genre of music. Findings from two additional samples who received musical stimuli from only a single genre (rock and jazz, respectfully) revealed that similar correlational patterns emerged. Further, these results were recoverable after controlling for sex differences and the Big Five personality traits. Hierarchical regression analyses showed the empathy consistently predicted musical preferences across samples and accounted for more unique variance than did personality for the Mellow and Intense music-preference dimensions.

Study 2 extended findings from Study 1 by examining how musical preferences were differentiated by empathizing-systemizing brain types. Type E preferred Mellow music and type S preferred Intense music. Analyses of detailed psychological attributes revealed that type E preferred music with low arousal, negative valence, and emotional depth. Type S preferred music with high arousal, and aspects of positive valence and cerebral depth. In terms of sonic attributes, type E preferred music with strings, while type S preferred music that was dense, distorted, loud, percussive, fast, and that featured brass and electric guitar. As in Study 1, the results in Study 2 remained significant after controlling for sex. These results confirmed our initial predictions that empathizers would prefer music from the Mellow dimension and systemizers would prefer music from the Intense dimensions. However, to our surprise, our prediction systemizers prefer music on the Sophisticated dimension was not supported. This prediction may only be applicable for either specified facets or extreme forms of systemizing, which is a topic to be investigated in further research.

### Future Directions

#### Neural correlates of musical preferences

Since there is evidence that differences in empathizing-systemizing have a neurobiological basis [45–47], findings from the present study suggest that individual differences in musical preferences may be linked to brain activity and structure. Advancements in neuroimaging technologies have helped researchers to explore the neurological basis of musical experience such as evoked emotion and structural processing [58–60]. However, researchers are only beginning to investigate the neurological underpinnings of musical preferences.

In one study, fMRIs of depressed patients and healthy controls found that listening to their favorite music was linked to activity in the medial orbital frontal cortex and nucleus accumbens (NAc), compared to a control group who listened to neutral music [61]. Specifically, activity in the left medial orbital cortex was positively associated with ratings of enjoyment when listening to their favorite music and the middle temporal cortex and NAc/ventral striatum was negatively associated with enjoyment ratings. In a study on musical tempo, preferences for musical tempo were correlated with the frequency of motor beta activity observed through EEG [62]. Future studies should extend this line of research by using the MUSIC model as a framework for which to conduct neural studies on musical preferences.

#### Extension to autism

The “Extreme Male Brain” theory (EMB) [34] is an extension of E-S theory and suggests that individuals with autism spectrum conditions (ASC) have below average levels of cognitive empathy alongside either intact or heightened levels of systemizing. Accordingly, individuals with ASC are typically classified as extreme type S [41, 43, 63]. Indeed, individuals with ASC experience music in ways that may be reflective of their hyper-systemizing and perceptual processing [64–69]. Yet little is known about the nature of musical preferences in autism.

A next step for research is to extend the findings from the present paper to extreme type S (individuals with autism). How do musical preferences in autism differ from typical developing populations? Are preferential patterns for extreme type S similar to those found for type S? Further, because autistic traits can be observed on a spectrum within the general population, future research could study the extent to which autistic traits (e.g. measured by the AQ), such as communication, social skills, imagination, and attention to detail, are manifested in musical preferences.

#### Can music increase empathy?

The present research identified the types of music that are linked to empathy, however, it did not examine if music can actually increase (or prime) empathy. That is, the results reported in this work are correlational and therefore causation cannot be inferred. However, given that previous research has found that group music-making can increase empathy [29] and prosocial behavior [70–72], it is reasonable to hypothesize that certain types of music may increase empathy more than others [36]. Future research should pinpoint the musical styles and specific sonic and psychological attributes that prime empathy, and also those elements that may decrease it. For example, based on results from Studies 1 and 2, one might hypothesize that music with emotional depth may increase empathy, whereas music with more strong and tense features may decrease it. Such information would be particularly useful for individuals with autism who report lower levels of cognitive empathy than the general population [73]. Findings from this line of research can be applied to music therapies, clinical interventions, and even computer-based interactive programs designed to teach emotions and mental states via music to individuals on the autistic spectrum. Importantly, studies on empathy priming through music can also extend to typically developing populations and help those who have difficulties with both outward and inward empathy. For example, for victims of violence who’s reported increased feelings of anger and aggression can lead to lower reflective functioning and mentalizing abilities [74].

## Conclusion

The present investigation extends theory and research on the determinants of musical preferences by examining its cognitive and affective basis. We overcame limitations that have hindered previous research on musical preferences by asking participants to report their preferential reactions to a variety of musical stimuli. This approach allowed us to observe preferences for nuanced musical attributes, which previous studies using genre-based methodologies have been unable to do. By employing the empathizing-systemizing theory, we identified the ways in which musical preferences are differentiated by cognitive ‘brain types’ and provide a framework for which future studies on autism and empathy priming can build from. Importantly, this is one of the first works to examine the links between musical behavior and systemizing, and raises important questions about the neurobiological basis of musical preferences.

## Supporting Information

S1 Data Dataset for Study 2. (ZIP)

S1 Fig Mean Preferences for the MUSIC Dimensions by Cognitive ‘Brain Type’ with Error Bars. (TIF)

S1 Table Summary of Sample Characteristics in Studies 1 and 2. (DOCX)

S2 Table Musical Excerpts Used in Studies 1 and 2. (DOCX)

S3 Table Partial Correlations Between Empathy, Systemizing, C/*D* scores and Musical Preferences while Controlling for Sex and Personality Traits. (DOCX)

S4 Table Subsampling Analysis for S2 in Study 1. (DOCX)

S5 Table Partial Correlations Between Empathy, Systemizing, *C*/*D* scores and Musical Preferences while Controlling for Sex and Personality Traits. (DOCX)

S6 Table Percentage of Participants with each Cognitive ‘Brain Type’ in Study 2 Compared to Previous Research. (DOCX)
