# Supplementary figures and images for: Musical Preferences are Linked to Cognitive Styles
Source: PLoS One. 2015 Jul 22;10(7):e0131151. doi: 10.1371/journal.pone.0131151 (PMC4511638; doi:10.1371/journal.pone.0131151)

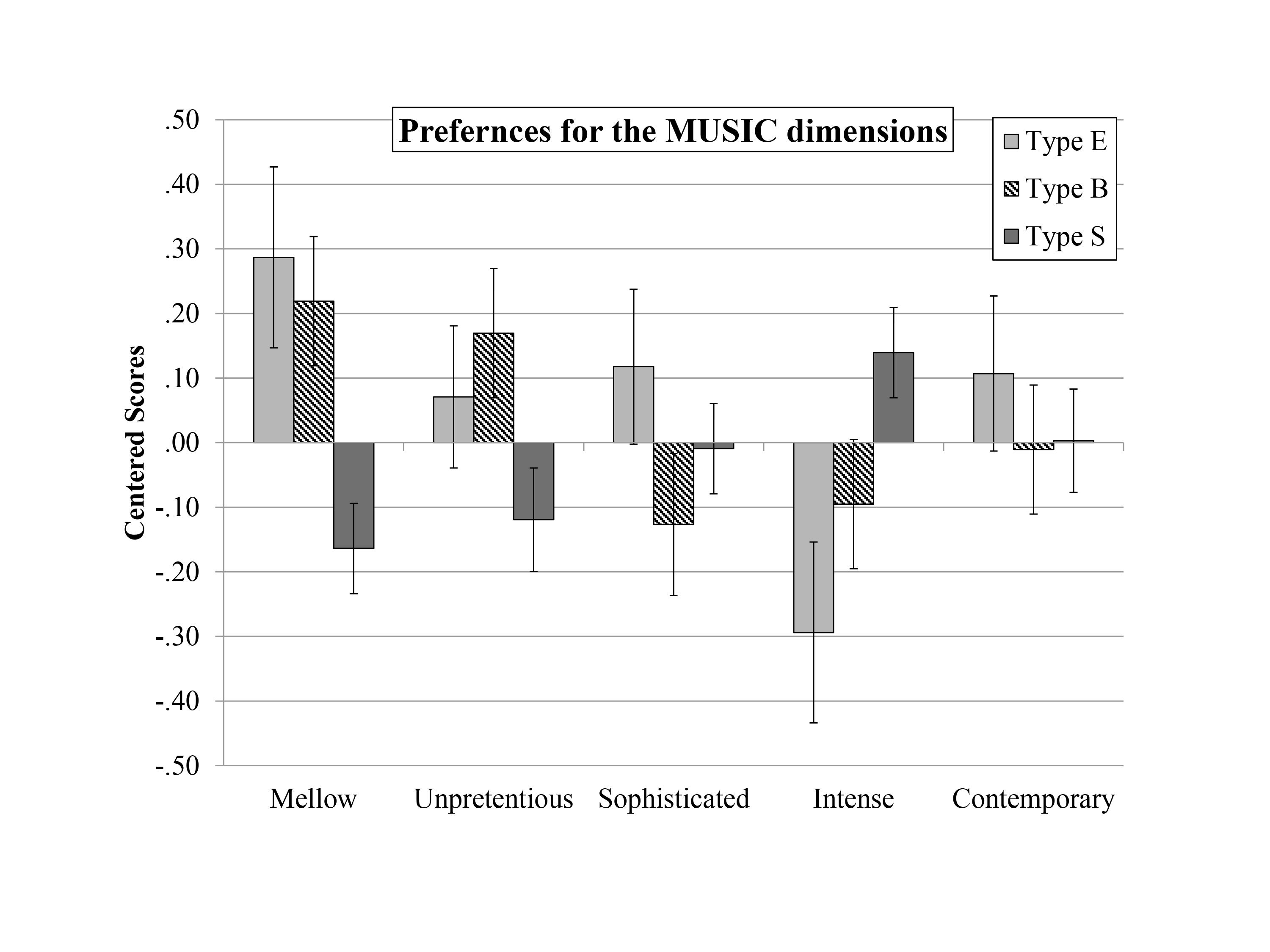

Supplement: S1 Fig — (TIF) [file pone.0131151.s002.tif]
