# Supplementary material for: Musical Preferences are Linked to Cognitive Styles
Source: PLoS One. 2015 Jul 22;10(7):e0131151. doi: 10.1371/journal.pone.0131151 (PMC4511638; doi:10.1371/journal.pone.0131151)
Supplement: S1 Table — (DOCX) [file pone.0131151.s003.docx]

**Table S1.** **Summary of Sample Characteristics in Studies 1 and 2.**

|  | **S1** | **S2** | **S3** | **S4** | **S5** |
| --- | --- | --- | --- | --- | --- |
| **Recruitment Method** | Facebook | Facebook | Facebook | Facebook | MTurk |
| **Type of Excerpts** | Mixed Genres | Mixed Genres | Rock Music | Jazz Music | Mixed Genres |
| **Number of Excerpts** | 50 | 25 | 50 | 50 | 25 |
| **Measures** | EQ | EQ | EQ | EQ | EQ, SQ-R |
|  | IPIP-NEO | IPIP-NEO | IPIP-NEO | IPIP-NEO |  |
| ***N*** | 2,178 | 891 | 747 | 320 | 353 |
| **% Female** | 60% | 63% | 60% | 57% | 62% |
| ***Mean* Age (*SD*)** | 24.80 (7.50) | 23.71 (6.47) | 25.31 (7.21) | 24.63 (7.45) | 31.10 (12.27) |

*Note*. S1 = Sample 1, S2 = Sample 2, S3 = Sample 3, S4 = Sample 4, S5= Sample 5. EQ = Empathy Quotient [26], SQ-R = Systemizing Quotient-Revised [41], IPIP-NEO = Proxy version of the NEO-PI-R [54] developed from the International Personality Item Pool [55]. Results from Samples 1 through 4 are reported in Study 1. Results from Sample 5 are reported in Study 2.
