# Supplementary material for: Musical Preferences are Linked to Cognitive Styles
Source: PLoS One. 2015 Jul 22;10(7):e0131151. doi: 10.1371/journal.pone.0131151 (PMC4511638; doi:10.1371/journal.pone.0131151)
Supplement: S2 Table — (DOCX) [file pone.0131151.s004.docx]

**Table S2. Musical Excerpts Used in Studies 1 and 2.**

| **Artist** | **Song** | **Genre** |
| --- | --- | --- |
| *Mixed Genre Excerpts*  *(administered to S1, S2, and S5*  *in Studies 1 and 2)* |  |  |
| AB+ | Recess^a^ | Electronica |
| Ali Handal | Sweet Scene^ab^ | Soft rock |
| Anglea Motter | Mama I’m Afraid to Go There^a^ | Bluegrass |
| Antonio Vivaldi | Concerto in C^a^ | Classical |
| Babe Gurr | Newsreel Paranoia^a^ | Bluegrass |
| Bankrupt | Face the Failure^ab^ | Punk |
| Benjamin Chan | MATRIX^a^ | Electronica |
| Bob Delevante | Penny Black^a^ | New country |
| Bruce Smith | Children of Spring^ab^ | Adult contemporary |
| Bruce Smith | Sonata A Major^ab^ | Classical |
| Carey Sims | Praying for Time^ab^ | Mainstream country |
| Ciph | Brooklyn Swagger^a^ | Rap |
| Cougars | Dick Dater^a^ | Classic rock |
| Curtis | Carrots & Grapes^ab^ | Rock-n-roll |
| Daniel Nahmod | I Was Wrong^ab^ | Traditional jazz |
| Dawn Over Zero | Out of Lies^a^ | Heavy metal |
| DJ Come of Age | Thankful^ab^ | R&B/soul |
| DNA | La Wally^ab^ | Classical |
| Exit 303 | Falling Down 2^ab^ | Classic rock |
| Five Finger Death Punch | Death Before Dishonor ^ab^ | Heavy metal |
| Five Finger Death Punch | White Knuckles^ab^ | Heavy metal |
| Five Foot Nine | Lana Marie^a^ | Country-rock |
| Frank Josephs | Mountain Trek^a^ | R&B/soul |
| Hillbilly Hellcats | That’s Not Rockabilly^ab^ | Rock-n-roll |
| Human Signals | Birth^a^ | Soft rock |
| James E. Burns | I’m Already Over You ^ab^ | New country |
| Kush | Sweet 5^a^ | Electronica |
| Language Room | She Walks^ab^ | Soft rock |
| Laura Hawthorne | Famous Right Where I Am^a^ | Mainstream country |
| Laurent Martin | Scriabin Etude Opus 65 No. 3^a^ | Avant-garde classical |
| Leo the Lionheart | Electro^a^ | Electronica |
| Lisa McCormick | Fernando Esta Feliz^a^ | Latin |
| Lisa McCormick | Let’s Love^ab^ | Adult contemporary |
| Ljova | Seltzer, do I Drink Too Much?^ab^ | Avant-garde classical |
| Magic Dingus Box | The Way It Goes^ab^ | Electronica |
| Moh Alileche | North Africa’s Destiny^a^ | World beat |
| Mykill Miers | Immaculate^ab^ | Rap |
| Paul Serrato & Co. | Who are You?^ab^ | Traditional jazz |
| Preston Middleton | Latin 4^a^ | R&B/soul |
| Robert LaRow | Sexy^ab^ | Europop |
| Sammy Smash | Get the Party Started^ab^ | Rap |
| Squint | Michigan^a^ | Punk |
| Straight Outta Junior High | Over Now^a^ | Punk |
| Taryn Murphy | Love Along The Way^a^ | Soft rock |
| The Cruxshadows | Go Away^a^ | Europop |
| The O'Neill Brothers | Through the Years^a^ | Quiet storm |
| The Stand In | Frequency of a Heartbeat^a^ | Punk |
| The Tomatoes | Johnny Fly^ab^ | Classic rock |
| Various Artists | La Trapera^ab^ | Latin |
| Walter Rodriguez | Safety^ab^ | Electronica |
| *Rock Excerpts*  *(administered to S3 in Study 1).* |  |  |
| Arcade Fire | Deep Blue | Rock |
| Beastie Boys | Sabotage | Rock |
| Beck | Guess I'm Doing Fine | Rock |
| Dave Mathews | Lover Lay Down | Rock |
| Death in Vegas Band | Girls | Rock |
| Frank Zappa | Inca Roads | Rock |
| Hall & Oates | One on One | Rock |
| Jeff Buckley | Dream Brother | Rock |
| Jeff Buckley | Hallelujah | Rock |
| Jeff Buckley | Parchman Farm Blues | Rock |
| Jeff Buckley | Eternal Life | Rock |
| Jeff Buckley | Kick Out the Jams | Rock |
| Led Zeppelin | Hot Dog | Rock |
| Led Zeppelin | Bron-Yr-Aur Stomp | Rock |
| Led Zeppelin | Boogie with Stu | Rock |
| Led Zeppelin | Black Mountain Side | Rock |
| Lynyrd Skynyrd | Gimmie Three Steps | Rock |
| Mark Ronson | L.S.F. | Rock |
| Ministry | Burning Inside | Rock |
| Ozomatli | Saturday Night | Rock |
| Phish | You Enjoy Myself | Rock |
| Pink Floyd | Comfortably Numb | Rock |
| Primus | Jerry was a Race Car Driver | Rock |
| Prince | When Doves Cry | Rock |
| Queen | The Invisible Man | Rock |
| Queen | Under Pressure | Rock |
| Queen | Crazy Little Thing Called Love | Rock |
| Queens of the Stone Age | Tension Head | Rock |
| Queens of the Stone Age | Battery Acid | Rock |
| Queens of the Stone Age | Quick & to the Pointless | Rock |
| Queens of the Stone Age | Misfit Love | Rock |
| Radiohead | No Surprises | Rock |
| Radiohead | Fake Plastic Trees | Rock |
| Radiohead | Reckoner | Rock |
| Radiohead | Back Drifts | Rock |
| Radiohead | Nude | Rock |
| Radiohead | Weird Fishes/Arpeggi | Rock |
| Radiohead | 15 Step | Rock |
| Radiohead | Body Snatchers | Rock |
| Radiohead | Electioneering | Rock |
| Rage Against the Machine | Bombtrack | Rock |
| Red Hot Chili Peppers | Higher Ground | Rock |
| Simon & Garfunkel | Kodachrome/Maybellene | Rock |
| The Beatles | Eleanor Rigby | Rock |
| The Beatles | Honey Don't | Rock |
| The Beatles | Act Naturally | Rock |
| The Doobie Brothers | Listen to the Music | Rock |
| The Police | Wrapped Around Your Finger | Rock |
| The RH Factor | Rich Man's Welfare | Rock |
| The Stooges | Down on the Street | Rock |
| *Jazz Excerpts*  *(administered to S4 in Study 1)* |  |  |
| Amos Easton (Bumble Bee Slim) | Ida Red | Jazz |
| Ben Sidran | Nothing Like The Sound Of Bebop | Jazz |
| Bessie Smith | The St. Louis Blues | Jazz |
| Billie Holiday | All of Me | Jazz |
| Bob James | Angela | Jazz |
| Bud Powell | Tempus Fugit (Tempus Fugue-it) | Jazz |
| Buddy Rich | The Nitty Gritty | Jazz |
| Cab Calloway & his Orchestra | Minnie The Moocher | Jazz |
| Candy Dulfer | Sax-A-Go-Goa | Jazz |
| Charlie Parker | Ko Ko | Jazz |
| Chet Atkins (& Merle Travis) | Nine Pound Hammer | Jazz |
| Derrick Shezbie | Softly As In A Morning Sunrise | Jazz |
| Django Reinhardt | Daphne | Jazz |
| Earl Klugh | Long Ago & Far Away | Jazz |
| Ella Fitzgerald & Louis Armstrong | Cheek To Cheek | Jazz |
| Gary Burton Quintet | Ictus/Syndrome | Jazz |
| Herb Albert | Rise | Jazz |
| Jacques Loussier | Italian Concerto: Presto | Jazz |
| Jacques Montagne | Charmaine | Jazz |
| Jazz Futures | Public Eye | Jazz |
| Jeff Beck | Blue Wind | Jazz |
| Joe Henderson | Junk Blues | Jazz |
| Joe Liggins & his Honeydrippers | The Honeydripper | Jazz |
| Joe Sample | In All My Wildest Dreams | Jazz |
| John Handy | Hard At Work | Jazz |
| Julian “Cannonball” Adderley | What Is This Thing Called Love? | Jazz |
| Kenny G | Forever In Love | Jazz |
| Kenny G | Sister Rose | Jazz |
| Lambert, Hendricks & Ross | Cloudburst | Jazz |
| Lidy Arbogast | Love to Love You Baby | Jazz |
| Louis Armstrong | Gut Bucket Blues | Jazz |
| Mezzoforte | After Hours | Jazz |
| Michael Civisca | Moonglow | Jazz |
| Michael Franks | My Foolish Heart | Jazz |
| Miles Davis | Directions I | Jazz |
| Miles Davis | John McLaughlin | Jazz |
| noJazz | Jungle Out | Jazz |
| noJazz | Pick Up | Jazz |
| Norah Jones | Come Away With Mea | Jazz |
| Patrick Saussois & Alma Sinti | Tune Up | Jazz |
| Paul Simon | Hobo's Blues | Jazz |
| Pharoah Sanders | Origin | Jazz |
| Pharoah Sanders | Moments Notice | Jazz |
| Room Eleven | One Of These Days | Jazz |
| Royal Crown Revue | Park's Place | Jazz |
| Sade | Smooth Operator | Jazz |
| St. Germain | Pont Des Arts | Jazz |
| St. Germain | Rose Rouge | Jazz |
| Stanley Clarke | Rock’n Roll Jelly | Jazz |
| Us3 | Cantaloop (Flip Fantasia) | Jazz |

*Note.* ^a^ *=* Indicates the excerpt was among the 50 administered to S1 of Study 1. ^b^ *=* Indicates the excerpt was among the 25 administered to S2 of Study 1 and S5 of Study 2.
