# Supplementary material for: Musical Preferences are Linked to Cognitive Styles
Source: PLoS One. 2015 Jul 22;10(7):e0131151. doi: 10.1371/journal.pone.0131151 (PMC4511638; doi:10.1371/journal.pone.0131151)
Supplement: S3 Table — (DOCX) [file pone.0131151.s005.docx]

**Table S3. Subsampling Analysis for S1 in Study 1.**

|  | **Empathy Quotient** | | | | |
| --- | --- | --- | --- | --- | --- |
|  | **S1** | **S1a** | **S1b** | **S1c** | **S1d** |
|  | **(*N*=2,178)** | **(*N*=300)** | **(*N*=400)** | **(*N*=500)** | **(*N*=600)** |
| **Mellow** | .09** | .10 | .17** | .14** | .07 |
| **Unpretentious** | .08** | .10 | .06 | .09* | .11* |
| **Sophisticated** | .03 | -.03 | .05 | .07 | .03 |
| **Intense** | -.10** | -.09 | -.16** | -.14** | -.09* |
| **Contemporary** | .04* | .04 | .08 | .02 | .02 |
| **Column Vector Correlations with S1** |  | .95 | .97 | .97 | .96 |

*Note*. Cell entries are correlations between musical preferences and scores on the Empathy Quotient. **p* < .05; ***p* < .01. S1 = Sample 1. S1a, S1b, S1c, and S2d are random subsamples of S1.
