# Supplementary material for: Musical Preferences are Linked to Cognitive Styles
Source: PLoS One. 2015 Jul 22;10(7):e0131151. doi: 10.1371/journal.pone.0131151 (PMC4511638; doi:10.1371/journal.pone.0131151)
Supplement: S4 Table — (DOCX) [file pone.0131151.s006.docx]

**Table S4. Subsampling Analysis for S2 in Study 1.**

|  | **Empathy Quotient** | | |
| --- | --- | --- | --- |
|  | **S2** | **S2a** | **S2b** |
|  | **(*N*=891)** | **(*N*=400)** | **(*N*=400)** |
| **Mellow** | .11** | .11* | .15** |
| **Unpretentious** | .04 | .05 | .05 |
| **Sophisticated** | .01 | .06 | .02 |
| **Intense** | -.11** | -.13** | -.14** |
| **Contemporary** | .09** | .09 | .08 |
| **Column Vector Correlations with S2** |  | .96 | .97 |

*Note*. Cell entries are correlations between musical preferences and scores on the Empathy Quotient. **p* < .05; ***p* < .01. S2 = Sample 2. S2a and S2b are random subsamples of S1.
