# Supplementary material for: Musical Preferences are Linked to Cognitive Styles
Source: PLoS One. 2015 Jul 22;10(7):e0131151. doi: 10.1371/journal.pone.0131151 (PMC4511638; doi:10.1371/journal.pone.0131151)
Supplement: S5 Table — (DOCX) [file pone.0131151.s007.docx]

**Table S5.** **Partial Correlations Between Empathy, Systemizing, *C*/*D* scores and Musical Preferences while Controlling for Sex and Personality Traits.**

|  | **Study 1** | | | | | | | | | | | | **Study 2** | | | | | | | |
| --- | --- | --- | --- | --- | --- | --- | --- | --- | --- | --- | --- | --- | --- | --- | --- | --- | --- | --- | --- | --- |
|  | **S1** | | | **S2** | | | **S3** | | | **S4** | | | **S5** | | | | | | | |
|  | **EQ** | **EQ.s** | **EQ.b5** | **EQ** | **EQ.s** | **EQ.b5** | **EQ** | **EQ.s** | **EQ.b5** | **EQ** | **EQ.s** | **EQ.b5** | **EQ** | **EQ.s** | **SQ** | **SQ.s** | ***C*score** | ***C*.s** | ***D*score** | ***D*.s** |
| ***MUSIC Dimensions*** |  |  |  |  |  |  |  |  |  |  |  |  |  |  |  |  |  |  |  |  |
| **Mellow** | .09** | .08** | .07** | .11** | .09* | .09* | .14** | .13** | .11** | .06 | .04 | .05 | .15** | .11* | -.12* | -.11* | .24** | .20** | -.24** | -.20** |
| **Unpretentious** | .08** | .07** | .07** | .04 | .02 | .03 | .04 | .02 | .02 | .01 | .00 | .03 | .12* | .11* | -.02 | -.02 | .13** | .11* | -.13** | -.11* |
| **Sophisticated** | .03 | .05* | .00 | .01 | .01 | .01 | .00 | .02 | -.02 | -.14* | -.10 | -.13* | .00 | -.03 | .07 | .07 | -.06 | -.09 | .06 | .09 |
| **Intense** | -.10** | -.10** | -.06** | -.11** | -.08* | -.09* | -.13** | -.11** | -.10* | -.08 | -.09 | -.07 | -.10 | -.06 | .08 | .07 | -.15** | -.12* | .15** | .12* |
| **Contemporary** | .04* | .03 | .03 | .09** | .09* | .09* | .13** | .09* | .13** | .11* | .11* | .08 | -.06 | -.04 | -.11* | -.11* | .04 | .06 | -.04 | -.06 |
| ***Psychological Attributes*** |  |  |  |  |  |  |  |  |  |  |  |  |  |  |  |  |  |  |  |  |
| **Arousal** |  |  |  |  |  |  |  |  |  |  |  |  |  |  |  |  |  |  |  |  |
| **High Arousal** | -.12** | -.11** | -.08** | -.11** | -.08* | -.09* | -.11** | -.10* | -.08* | -.11* | -.10 | -.11 | -.12* | -.08 | .08 | .07 | -.17** | -.13* | .17** | .13* |
| **Low Arousal** | .11** | .11** | .07** | .11** | .08* | .09* | .15** | .14** | .13** | .09 | .07 | .08 | .13* | .08 | -.09 | -.08 | .19** | .15** | -.19** | -.15** |
| **Valence** |  |  |  |  |  |  |  |  |  |  |  |  |  |  |  |  |  |  |  |  |
| **Positive Valence** | -.07** | -.04* | -.07** | -.05 | -.02 | -.05 | -.02 | -.03 | -.03 | -.05 | -.03 | -.05 | -.16** | -.12* | .06 | .05 | -.19** | -.15** | .19** | .15** |
| **Negative Valence** | .07** | .04 | .07** | .08* | .04 | .07* | .14** | .13** | .12** | .07 | .04 | .06 | .21** | .16** | -.05 | -.04 | .24** | .18** | -.24** | -.18** |
| **Depth** |  |  |  |  |  |  |  |  |  |  |  |  |  |  |  |  |  |  |  |  |
| **Emotional Depth** | .11** | .11** | .08** | .09** | .07 | .09* | .16** | .14** | .14** | .11* | .09 | .10 | .12* | .08 | -.07 | -.06 | .17** | .13** | -.17** | -.13* |
| **Cerebral Depth** | .03 | .04 | .01 | -.01 | -.04 | .00 | .05 | .05 | .01 | -.09 | -.07 | -.06 | .11* | .07 | .07 | .09 | .04 | -.01 | -.04 | .01 |
| ***Sonic Attributes*** |  |  |  |  |  |  |  |  |  |  |  |  |  |  |  |  |  |  |  |  |
| **Acoustic Features** |  |  |  |  |  |  |  |  |  |  |  |  |  |  |  |  |  |  |  |  |
| **Dense** | -.12** | -.11** | -.08** | -.10** | -.06 | -.09* | -.09* | -.06 | -.08 | -.03 | -.05 | -.06 | -.14* | -.11* | .06 | .06 | -.18** | -.15** | .18** | .15** |
| **Distorted** | -.13** | -.12** | -.09** | -.12** | -.09* | -.11** | -.12** | -.10** | -.09* | -.03 | -.04 | -.03 | -.10 | -.06 | .10 | .09 | -.18** | -.13* | .18** | .13* |
| **Electric** | -.07** | -.07** | -.06** | -.05 | -.02 | -.10** | -.10** | -.08 | -.09* | .05 | .04 | -.02 | -.12* | -.09 | .02 | .01 | -.13* | -.10* | .13* | .10* |
| **Heavy bass** | -.08** | -.08** | -.06** | .03 | .04 | .04 | -.11** | -.10* | -.09* | .08 | .08 | .06 | -.07 | -.04 | -.02 | -.03 | -.05 | -.02 | .05 | -.02 |
| **Instrumental** | -.07** | -.05* | -.06** | .01 | -.01 | .00 | -.11** | -.07 | -.12** | -.18** | -.16** | -.13* | .12* | .10 | .05 | .06 | .07 | .04 | -.07 | -.04 |
| **Loud** | -.12** | -.11** | -.08** | -.11** | -.08* | -.09* | -.14** | -.12** | -.11** | -.06 | -.05 | -.07 | -.12* | -.08 | .08 | .07 | -.18** | -.14** | .18** | .14** |
| **Percussive** | -.11** | -.11** | -.07** | -.09** | -.06 | -.07 | -.12** | -.10** | -.10* | -.01 | -.01 | -.04 | -.14** | -.11* | .05 | .04 | -.17** | -.14* | .17** | .14** |
| **Tempo** | -.14** | -.13** | -.10** | -.11** | -.08* | -.09* | -.15** | -.14** | -.13** | -.08 | -.05 | -.09 | -.13* | -.09 | .08 | .07 | -.19** | -.15** | .19** | .15** |
| **Instrumental Features** |  |  |  |  |  |  |  |  |  |  |  |  |  |  |  |  |  |  |  |  |
| **Acoustic guitar** | .11** | .10** | -.04* | .02 | .01 | -.04 | .08* | .06 | -.07 | -.05 | -.06 | .02 | .10 | .10 | -.03 | -.03 | .12* | .12* | -.12* | -.12* |
| **Bass guitar** | -.10** | -.09** | -.07** | -.06 | -.03 | -.03 | -.12** | -.10** | -.10* | .06 | .07 | .04 | -.14** | -.12* | .06 | .05 | -.18** | -.16** | .18** | .16** |
| **Brass** | .04 | .06** | .01 | -.07* | -.04 | -.08* | -.02 | -.01 | -.01 | -.02 | -.01 | .01 | -.08 | -.06 | .02 | .01 | -.09 | -.06 | .09 | -.06 |
| **Cymbals** | -.10** | -.09** | -.06** | -.09** | -.07 | -.06 | -.12** | -.09* | -.08 | -.05 | -.04 | -.08 | -.09 | -.07 | .04 | .04 | -.12* | -.10 | .12* | .10 |
| **Drum set** | -.11** | -.10** | -.06** | -.08* | -.06 | -.05 | -.15** | -.13** | -.13** | -.08 | -.08 | -.09 | -.12* | -.11* | .09 | .09 | -.19** | -.18** | .19** | .18** |
| **Electric guitar** | -.10** | -.10** | -.06** | -.12** | -.09* | -.10** | -.11** | -.10** | -.09* | -.02 | -.04 | -.02 | -.18** | -.16** | .10 | .09 | -.25** | -.23** | .25** | .23** |
| **Piano** | .02 | .03 | .00 | .02 | .02 | .02 | -.03 | -.02 | -.04 | -.04 | .00 | -.05 | -.11* | -.11* | .05 | .05 | -.14** | -.14** | .14** | .14** |
| **Raspy voice** | -.10** | -.10** | -.06** | -.07* | -.07* | -.06* | -.09* | -.09* | -.06 | .15** | .13* | .12* | .01 | .02 | .06 | .06 | -.04 | -.03 | .04 | -.03 |
| **Strings** | .02 | .03 | .00 | .03 | .00 | .04 | .08* | .06 | .05 | -.08 | -.08 | -.05 | .14** | .13* | -.05 | -.04 | .17** | .16** | -.17** | -.16** |
| **Synthesizer** | .03 | .02 | .01 | .00 | .00 | .01 | .07 | .07 | .09* | .09 | .08 | .06 | .00 | .00 | -.01 | -.01 | .01 | .01 | -.01 | .01 |
| **Woodwinds** | .02 | .03 | .00 | -.02 | -.03 | -.03 | -.02 | .00 | -.03 | -.07 | -.07 | -.07 | .16** | .11* | .10 | .11* | .07 | .02 | -.07 | -.02 |
| **Yelling voice** | -.08** | -.09** | -.05* | -.03 | -.03 | -.04 | -.10** | -.10* | -.07 | .10 | .08 | .08 | .02 | .02 | .06 | .06 | -.03 | -.03 | .03 | -.03 |

*Note:* Cell entries are partial correlations between musical preferences and empathy, systemizing and *C*/*D* scores while controlling separately for sex and the Big Five personality traits. S1 = Sample 1, S2 = Sample 2, S3 = Sample 3, S4 = Sample 4, S5 = Sample 5. EQ = Empathy Quotient [26], SQ-R = Systemizing Quotient-Revised [41]. “.s” = controlling for sex, “.b5” = controlling for the Big Five personality traits. *Ns* = 2,178 (S1), 891 (S2), 747 (S3), 320 (S4), and 353 (S5). **p* < .05; ***p* < .01.
