# Supplementary material for: Musical Preferences are Linked to Cognitive Styles
Source: PLoS One. 2015 Jul 22;10(7):e0131151. doi: 10.1371/journal.pone.0131151 (PMC4511638; doi:10.1371/journal.pone.0131151)
Supplement: S6 Table — (DOCX) [file pone.0131151.s008.docx]

| **Table S6.** **Percentage of Participants with each Cognitive ‘Brain Type’ in Study 2 Compared to Previous Research.** | | | | |
| --- | --- | --- | --- | --- |
|  | **Males** | **Males*** | **Females** | **Females*** |
| **Extreme Type E** | 0 | 0.1 | 0 | 4.3 |
| **Type E** | 7.9 | 15.1 | 22.6 | 44.8 |
| **Type B** | 16.5 | 30.3 | 37.8 | 29.3 |
| **Type S** | 75.6 | 49.5 | 39.6 | 20.7 |
| **Extreme Type S** | 4.5 | 5.0 | 1.4 | 0.9 |

*Note*: * = data from Wheelwright et al. [41].
